# Next

**Published:** 1886-04

**Authors:** 


### NEXT.

A Polyclinic in Atlanta, Ga., is announced in the *Atlanta Medical and Surgical Journal for April*, but no details are given, except that "it is proposed to organize and systematise the medical out-door work of Atlanta,'' and that ''there will be six departments presided over by'' Drs. So and so. An item of so much importance should be explicit. *How* ''organize and systematise the medical out-door work of Atlanta?'' That is an item we would like to catch on to, over here in Texas.

